# Complex venous access in superior vena cava syndrome secondary to gray zone lymphoma, case report

**DOI:** 10.3389/fonc.2026.1811919

**Published:** 2026-09-16

**Authors:** Martin Razus, Maros Kunderlik, Michal Marko, Vanda Repiska, Pavol Povinec, Bela Mrinakova

**Affiliations:** 1 Institute of Medical Biology, Genetics and Clinical Genetics, Faculty of Medicine, Comenius University, Bratislava, Slovakia; 2 Department of Oncohematology, Faculty of Medicine, Comenius University, Bratislava, Slovakia; 3 National Cancer Institute, Bratislava, Slovakia; 4 I. Department of Oncology, Faculty of Medicine, Comenius University, Bratislava, Slovakia; 5 St. Elisabeth Cancer Institute, Bratislava, Slovakia; 6 BIONT, Bratislava, Slovakia

**Keywords:** catheter-related infection, central venous access, femoral catheterization, mediastinal gray zone lymphoma, superior vena cava

## Abstract

**Introduction:**

Mediastinal gray zone lymphoma (MGZL) is a rare and biologically complex lymphoma with overlapping features of primary mediastinal B-cell lymphoma and classical Hodgkin lymphoma. Bulky mediastinal disease frequently complicates central venous access, particularly in the presence of superior vena cava (SVC) syndrome. Reports addressing optimal vascular access strategies in this setting remain limited.

**Case presentation:**

A 41-year-old man presented with newly diagnosed MGZL and a large mediastinal mass causing clinically significant SVC obstruction. The patient required urgent initiation of intensive systemic treatment; however, standard central venous access was contraindicated due to altered thoracic venous anatomy. A tunneled femoral catheter was therefore inserted to allow treatment initiation with rituximab plus dose-adjusted etoposide, doxorubicin, vincristine, cyclophosphamide, and prednisone (R-DA-EPOCH). The clinical course was complicated by catheter-related bloodstream infections caused by multidrug-resistant pathogens, septic shock, and pulmonary embolism, necessitating intensive care management. Following partial tumor response and anatomical decompression, duplex ultrasound–guided evaluation enabled successful implantation of a chest port via the left internal jugular vein, during which a duplicated superior vena cava was identified. Subsequent catheter-tip thrombosis was managed with low-molecular-weight heparin. Through coordinated multidisciplinary care, full-intensity R-DA-EPOCH was completed up to dose level 4, resulting in complete metabolic remission.

**Conclusion:**

This case highlights the critical importance of individualized venous access strategies in patients with mediastinal lymphoma complicated by SVC syndrome. Imaging-guided vascular assessment, multidisciplinary coordination, and adherence to international and national guidelines can enable safe delivery of curative-intent intensive chemotherapy, even in the setting of severe anatomical distortion and life-threatening complications. The case offers a visually documented, practical framework for managing venous access in complex anatomical conditions associated with superior vena cava syndrome.

## Introduction

Mediastinal gray zone lymphoma (MGZL) is recognized in the WHO 5th edition classification as a mediastinal B-cell lymphoma with overlapping features of primary mediastinal large B-cell lymphoma and classical Hodgkin lymphoma. The diagnosis is currently restricted to mediastinal cases, whereas morphologically similar lymphomas presenting outside the mediastinum are classified as diffuse large B-cell lymphoma, not otherwise specified (DLBCL, NOS). Patients typically present with bulky mediastinal masses and superior vena cava syndrome, which may complicate central venous access. Although temporary femoral access can be used, durable venous access is essential for intensive regimens such as rituximab plus dose-adjusted etoposide, doxorubicin, vincristine, cyclophosphamide, and prednisone (DA-EPOCH-R). Prognosis of MGZL remains inferior compared with PMBL or classical Hodgkin lymphoma, underscoring the importance of maintaining full treatment intensity.

## Case report

A 41-year-old man with morbid obesity (BMI 46) without significant past medical history or known congenital vascular abnormalities, presented with a three-week history of progressively worsening dyspnea, facial and upper-body swelling, dysphagia, and prominent dilated veins over the left side of the neck. PET/CT revealed a massive mediastinal tumor infiltrating the superior vena cava and adjacent structures (**Figure 1**). The biopsy confirmed MGZL with weak CD20 expression. Urgent initiation of intensive systemic therapy was required; however, access through the superior vena cava system was not considered feasible or safe. The patient had complete tumor-related obstruction of the SVC, thrombosis of the right internal jugular and subclavian veins, and a presumed patent left-sided duplicated SVC with uncertain cardiac drainage. In this anatomical and thrombotic context, upper-body central venous catheterization would have carried a high risk of malposition, inadequate venous drainage, and further thrombotic or mechanical complications. As endovascular thrombectomy was not indicated, therapeutic anticoagulation with dalteparin (capped at 18, 000 IU daily) was initiated, and a tunneled femorally inserted central catheter (FICC) was selected as the most appropriate temporary central venous access to allow prompt initiation of DA-EPOCH-R, a regimen requiring reliable central access because of its prolonged continuous infusion and vesicant components. A taurolidine-based catheter lock solution was routinely used as part of the institutional protocol for the prevention of catheter-related bloodstream infections. Ten days following the first cycle of DA-EPOCH-R, the patient was admitted directly to the intensive care unit with septic shock secondary to polymicrobial catheter-related bloodstream infection, with blood cultures positive for Escherichia coli, Klebsiella aerogenes, and Klebsiella pneumoniae, together with Pseudomonas aeruginosa pneumonia. The clinical course was further complicated by pulmonary embolism. During this acute episode, tunnel infection of the femoral inserted central catheter (FICC) was diagnosed, necessitating catheter removal after 11 days *in situ*.

**Figure 1:**
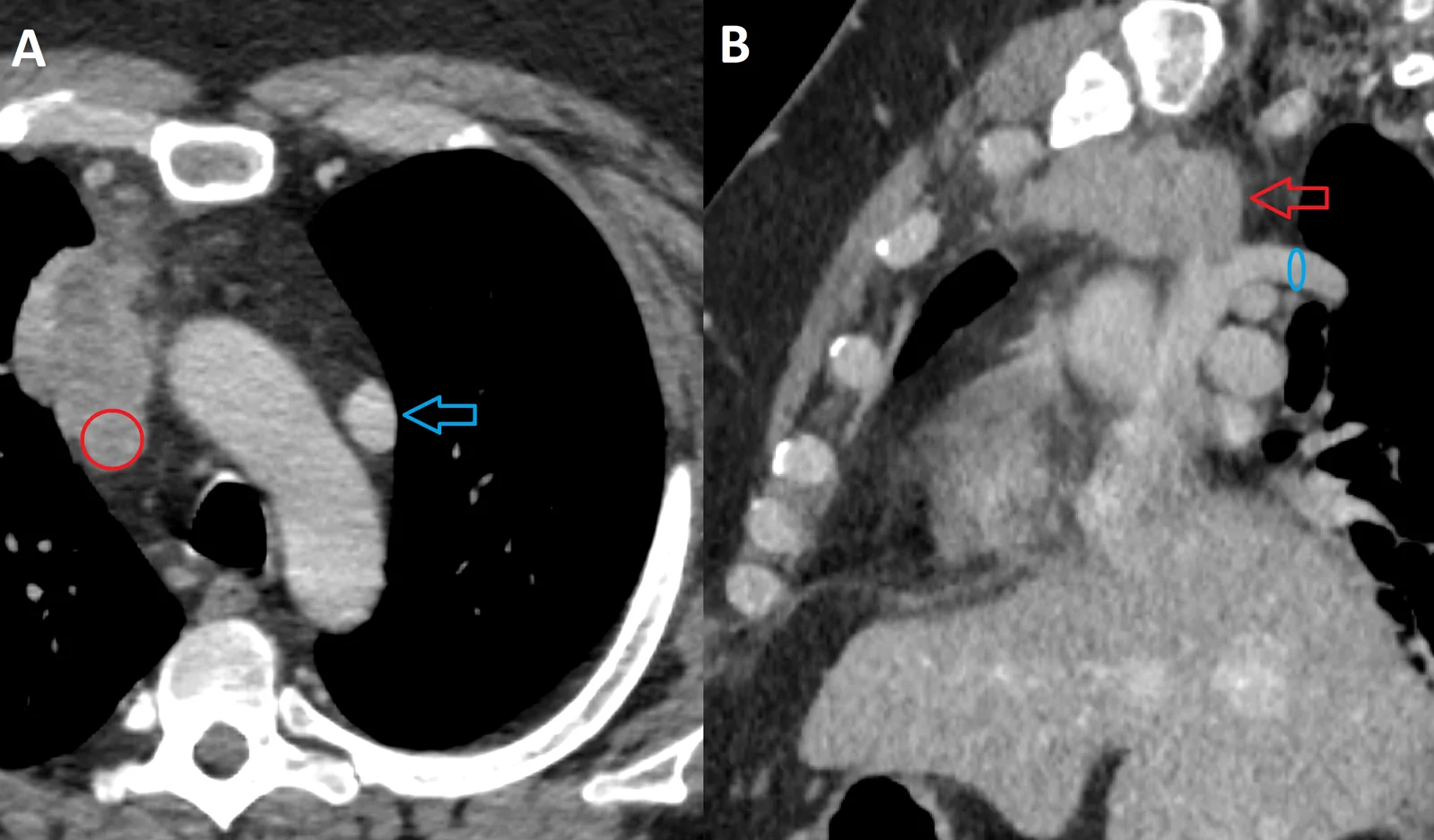
**(A)** Axial CT image demonstrating occlusion of the right superior vena cava by tumor infiltration (red circle). The size of the circle corresponds to the expected caliber of the superior vena cava and correlates with the lumen diameter in its uninvolved segment. A persistent left-sided duplicated superior vena cava is indicated by the blue arrow. (Contrast administered via a peripheral venous cannula in the left cubital vein). **(B)** Sagittal CT image from the same examination demonstrating the longitudinal extent of the infiltrative superior vena cava occlusion (red arrow). Distal to the level of infiltration, recanalization of the superior vena cava is visible, predominantly due to venous inflow from the azygos vein (blue circle). (Contrast administered via a peripheral venous cannula in the left cubital vein).

After resolution of the infection and CT confirmation of partial tumor response with mediastinal decompression, a 6.6F chest port was successfully implanted via the left internal jugular vein under real-time ultrasound and ECG guidance.

The procedure was technically challenging. Although the duplicated SVC had already been identified on the initial CT examination, its anatomical course became more clearly delineated on post-procedural imaging following partial regression of the mediastinal mass and contrast administration through the implanted left jugular venous access device (**Figure 2**).

**Figure 2:**
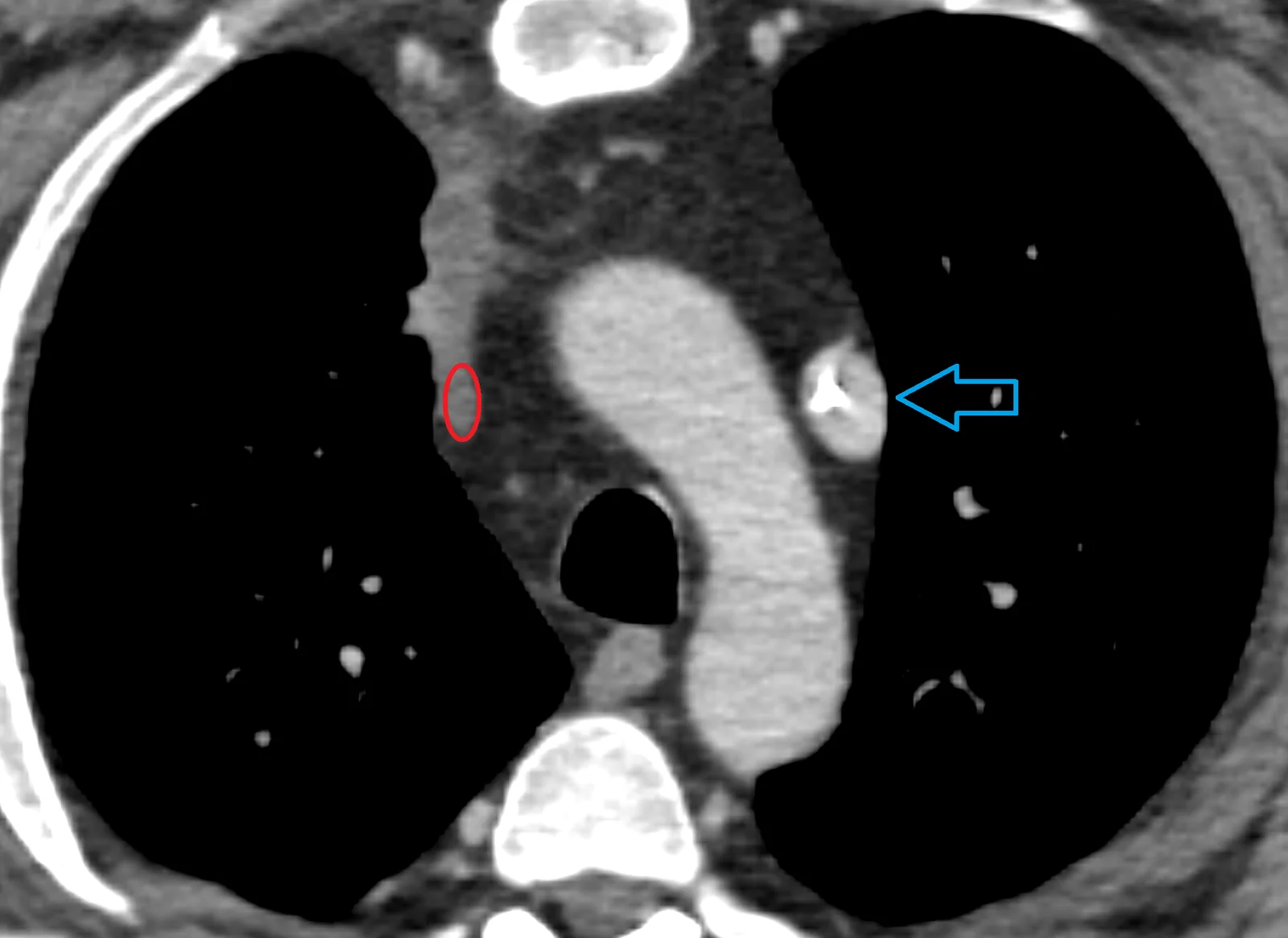
CT image after first cycle of DA-EPOCH-R demonstrating regression of the previously identified pathological infiltrative lesion, with persistent deformity of the lumen of the right superior vena cava caused by residual tumor infiltration (red circle). The course of the indwelling catheter within the left-sided duplicated superior vena cava is indicated by the blue arrow. (Contrast administered via the implanted left jugular venous access device).

After three cycles of chemotherapy, PET/CT confirmed partial remission, with an incidental finding of catheter-tip thrombosis, possibly consistent with a fibrin sheath (**Figure 3**).

**Figure 3:**
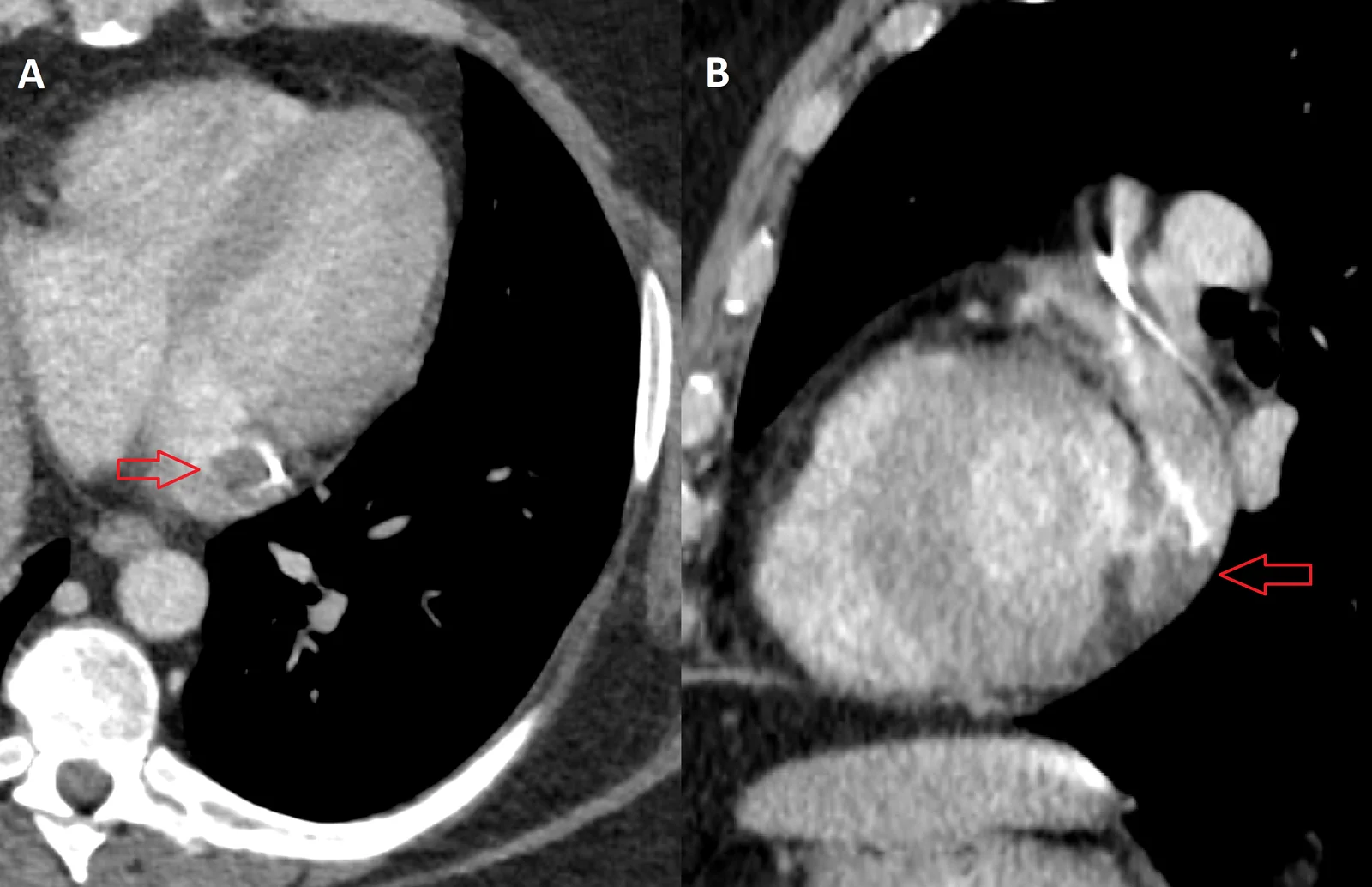
**(A)** Axial CT image demonstrating a hypodense contrast-filling defect adjacent to the tip of the catheter, which terminates in the right atrium, consistent with an adherent mural thrombus (red arrow). (Contrast administered via the implanted left jugular venous access device). **(B)** Corresponding sagittal CT image demonstrating the approximate extent of the mobile (floating) thrombus attached to the catheter tip within the right atrium (red arrow). (Contrast administered via the implanted left jugular venous access device).

Through coordinated multidisciplinary management, the patient successfully completed six cycles of DA-EPOCH-R with dose escalation to level 4, achieving complete metabolic remission and complete resolution of symptoms related to both SVC syndrome and pulmonary embolism.

## Discussion

This case highlights the significant challenges of securing central venous access in patients with mediastinal gray zone lymphoma complicated by SVC syndrome, in whom standard upper-body access may be anatomically contraindicated. International recommendations from the ESMO Guidelines Committee identify tunneled central venous catheters or implantable ports as the preferred devices for prolonged systemic therapy, whereas femoral access should generally be avoided because of higher risks of infection and thrombosis, except in situations such as SVC obstruction or severe mediastinal venous compromise (1). Reliable venous access is critical for delivery of vesicant chemotherapy and supportive care. Current clinical evidence reinforces that totally implantable venous access ports (TIVAPs) and implantable chest ports remain the preferred devices for prolonged chemotherapy, as they are associated with significantly lower risks of infection, occlusion, phlebitis, and dislodgement when compared with peripherally inserted central catheters (PICCs) in patients with hematologic malignancies undergoing systemic therapy (2). Conversely, PICCs have practical advantages such as ease of insertion and long durability and have been supported as a safe option with low complication rates in well-controlled lymphoid malignancy cohorts (3). Although femoral central venous access is generally avoided when upper-body access is feasible, it represents an accepted alternative in patients with obstruction or thrombosis of the SVC system. FICCs have been described as a feasible approach for chemotherapy administration, particularly when treatment cannot be delayed and peripheral venous access is unsuitable for continuous vesicant therapy. In the present case, complete tumor-related obstruction of the superior vena cava, thrombosis of the right internal jugular and subclavian veins, and uncertain drainage of the presumed duplicated left superior vena cava precluded conventional upper-body central venous access. Furthermore, severe SVC syndrome with markedly impaired upper-body venous return rendered peripheral venous access unreliable for prolonged infusion of vesicant chemotherapy. Under these circumstances, a tunneled femoral inserted central catheter provided the most appropriate vascular access until definitive chest port implantation became feasible.

Infection prevention remains a cornerstone of management, particularly in immunocompromised patients. Evidence-based recommendations emphasize strict aseptic technique during insertion and maintenance, including chlorhexidine-based skin antisepsis and meticulous hub disinfection, and, where appropriate, antimicrobial catheter lock solutions such as taurolidine to reduce the incidence of catheter-related bloodstream infections (4). These measures are especially relevant when alternative access routes must be used or when catheter dwell time is prolonged.

Device selection must therefore balance urgency of treatment, anatomical feasibility, and complication risk. Regional expert recommendations similarly support ports or tunneled catheters for therapies expected to exceed 30 days and consider femoral access a last-line strategy requiring intensive monitoring (5). Emerging clinical data indicate that although upper-body devices remain preferable, alternative strategies such as femorally inserted tunneled central catheters can provide a viable bridge when thoracic venous pathways are obstructed, though complication rates may be higher and careful patient selection is essential (6). Together, these findings support an individualized, anatomy-adapted approach to vascular access selection (**Table 1**).

**Table 1:** Evidence-based recommendations for central venous access selection in oncology patients.

Clinical scenario	Preferred vascular access	Key considerations	Level of evidence
Intermediate-duration therapy (weeks–6 months)	Peripherally inserted central catheter (preferably tunneled)	Suitable for outpatient therapy; low insertion risk; acceptable complication rates with proper maintenance	II–III
Long-term or high-frequency therapy	Tunneled central venous catheter or totally implantable venous access port	Standard for prolonged therapy; lowest long-term infection and thrombosis risk	I–II
Superior vena cava obstruction or no upper-body access	Femorally inserted central catheter or tunneled femoral catheter (temporary strategy)	Last-line option when thoracic access is not feasible; increased infection and thrombosis risk; requires close monitoring	II–III
Catheter insertion and handling (all devices)	Ultrasound-guided insertion with intracavitary ECG tip confirmation and strict aseptic technique	Reduces mechanical and infectious complications; saline flush preferred; minimize routine heparin locking	I

Levels of evidence: I = meta-analyses or randomized controlled trials; II = prospective studies; III = retrospective/observational studies; IV = expert consensus or guideline-based recommendations.

Patients with bulky mediastinal lymphoma receiving intensive chemotherapy are at particularly high risk of infectious complications because of disease burden, treatment-related cytopenias, and the need for prolonged central venous access. In our case, the femorally inserted central catheter was complicated by tunnel infection and catheter-related bloodstream infection after 11 days *in situ*. This experience raises the question of whether, an initially implanted femorally inserted central venous port (FIVAP) rather than a tunneled femoral catheter might represent an alternative strategy with potentially lower infection risk.

Therapeutic anticoagulation in patients with cancer-associated thrombosis and morbid obesity remains challenging because robust evidence in this population is limited. In the landmark CLOT trial (7), dalteparin was administered at 200 IU/kg once daily during the first month, with a maximum daily dose of 18, 000 IU. However, more recent international guidelines recommend therapeutic low-molecular-weight heparin (LMWH) dosing according to actual body weight rather than routine dose capping in obese patients, although these recommendations were not developed specifically for patients with cancer-associated thrombosis receiving intensive chemotherapy (8). Consideration of anti-factor Xa LMWH dose monitoring may be appropriate in selected cases of extreme body weight. In patients receiving intensive chemotherapy, anticoagulation management is further complicated by treatment-induced thrombocytopenia, requiring continuous reassessment of the balance between thrombotic and bleeding risks.

In addition, thoracic venous anomalies may complicate procedural planning. Variants such as duplicated SVC, which may remain clinically silent, can affect catheter placement, tip positioning, and hemodynamics and should therefore be considered during imaging assessment and intervention (9).

Therapeutically, MGZL remains an aggressive entity requiring full-intensity combination therapy. Prospective clinical data demonstrate that DA-EPOCH-R provides superior outcomes compared with historical regimens such as R-CHOP or ABVD, supporting its use as a preferred frontline approach despite greater logistical complexity (10).

## Conclusion

This report has several limitations inherent to its single-case design, and the management described reflects an individualized multidisciplinary approach tailored to highly complex anatomical and clinical circumstances. Nevertheless, it highlights the importance of prompt referral to an experienced multidisciplinary center, where complex vascular access strategies can be implemented without delaying curative-intent therapy. Although alternative vascular access approaches may carry a higher risk of infectious and thrombotic complications, timely initiation and uninterrupted delivery of intensive systemic treatment should remain the primary priority, supported by close monitoring and appropriate supportive care.

Despite life-threatening complications, including polymicrobial bloodstream infection, septic shock, thrombosis, and pulmonary embolism, the patient ultimately completed full-dose therapy and achieved complete metabolic remission. This outcome underscores the importance of coordinated multidisciplinary management integrating hematology, interventional radiology, infectious disease, vascular medicine, and critical care. Such collaboration is often decisive in enabling delivery of optimal oncologic therapy in anatomically and clinically challenging mediastinal presentations.

## Data Availability

The original contributions presented in the study are included in the article/supplementary material. further inquiries can be directed to the corresponding authors.
